# Efficacy of concurrent chemoradiotherapy for patients with limited-disease small-cell lung cancer: a retrospective, nationwide, population-based cohort study

**DOI:** 10.1186/s12885-021-08082-2

**Published:** 2021-03-31

**Authors:** Seo Ree Kim, Ji Hyung Hong, Soo-Yoon Sung, Yeo Hyung Kim, Sang Hoon Chun, Hyun Woo Lee, Jung Soo Lee, Yoon Ho Ko

**Affiliations:** 1 Division of Oncology, Department of Internal Medicine, College of Medicine, The Catholic University of Korea, 222, Banpo-daero, Seocho-gu, Seoul, 06591 Republic of Korea; 2 Department of Radiation Oncology, College of Medicine, The Catholic University of Korea, Seoul, Republic of Korea; 3 Department of Rehabilitation Medicine, College of Medicine, The Catholic University of Korea, Seoul, Republic of Korea; 4 Department of Hematology-Oncology, Ajou University School of Medicine, Suwon, Republic of Korea; 5 Cancer Research Institute, College of Medicine, The Catholic University of Korea, 222, Banpo-daero, Seocho-gu, Seoul, 06591 Republic of Korea

**Keywords:** Cohort study, Chemoradiotherapy, Efficacy, Small-cell lung carcinoma

## Abstract

**Background:**

Small-cell lung cancer (SCLC) is a highly proliferative, rapidly growing tumor with a poor prognosis, even in cases of limited disease (LD). Timely and accurate high-intensity therapy is necessary. For concurrent chemoradiotherapy (CCRT), etoposide/platinum (EP)-based regimens are recommended, although irinotecan/platinum (IP)-based regimens are also effective with radiotherapy. This large-scale, retrospective, nationwide cohort study aimed to analyze the efficacy of CCRT in patients with LD-SCLC.

**Methods:**

Population data registered between January 2008 and December 2018 was extracted from the Health Insurance Review and Assessment Service of Korea database. Survival outcomes of 4446 LD-SCLC patients who received CCRT were analyzed.

**Results:**

Patients who received EP-CCRT (*n* = 4187) showed better time to first subsequent therapy (TFST: 11.2 months) and overall survival (OS: 22.2 months) than those who received IP-CCRT (*n* = 259; TFST: 9.6 months, *P* = 0.0477; OS: 16.4 months, *P* <  0.0001). When CCRT failed, dual-agent chemotherapy (*n* = 925; OS: 9.1 months) provided a better survival benefit than single-agent chemotherapy (*n* = 815; OS: 7.5 months). IP-based chemotherapy resulted in better OS (9.6 months) than EP-based chemotherapy (7.1 months, *P* = 0.017) in platinum-resistant relapsed patients; the opposite was observed for platinum-sensitive relapsed patients (OS: EP, 17.2 months; IP, 6.6 months; *P* <  0.0001). Poisson regression analysis demonstrated that age, EP-CCRT, and hypercholesterolemia retained significant associations with OS after adjustment for all variables.

**Conclusion:**

In the Korean population, the effects of EP-CCRT on OS and TFST are significantly more favorable than those of IP-CCRT.

**Supplementary Information:**

The online version contains supplementary material available at 10.1186/s12885-021-08082-2.

## Background

Small-cell lung cancer (SCLC) is an aggressive lung cancer subtype that accounts for only 12–15% of all lung cancer diagnoses [1, 2]. At diagnosis, approximately one third of patients have limited-disease (LD) SCLC. The limited-stage disease is confined to the ipsilateral hemithorax or mediastinal or supraclavicular lymph nodes, which can be safely encompassed within a radiation field, while metastatic tumors are categorized as extensive-disease (ED) SCLC [1]. For LD-SCLC, the median survival and 2-year survival rates have been reported to be 15–20 months and 20–40%, respectively. Importantly, the proportion of patients who survive for 5 years is only 14–26% [2]. Although LD-SCLC is a potentially curable disease, it exhibits a high histological proliferation rate and clinically varied manifestations, such as paraneoplastic syndrome. Therefore, any delay can result in a change in the treatment strategy and worsen the prognosis [2, 3].

Almost two decades ago, the addition of twice-daily radiotherapy to combination chemotherapeutic agents was the cornerstone of LD-SCLC treatment [4, 5], a strategy that significantly improved the survival of patients with LD-SCLC. In the era of immunotherapy, there has been a paradigm shift in the treatment of SCLC [6, 7]; however, concurrent chemoradiotherapy (CCRT) remains the current standard treatment worldwide [2, 3]. In previous studies, the overall response rate after CCRT reached ≥80%, with a complete remission rate of up to 45% [8, 9]. A large proportion of patients died from recurrence and distant metastasis. These outcomes resulted in an unmet need for the development of chemotherapeutic agents and combinations that exhibited greater antitumor effects and superior radiosensitization. CCRT based on an etoposide/platinum (EP)-based regimen has been the standard protocol since the early 1990s. In addition, an irinotecan/platinum (IP)-based regimen with radiation treatment has been reported to be effective and tolerable for patients with untreated LD-SCLC [10, 11]. Although several studies have verified the effectiveness of these two regimens in cases of LD-SCLC with similar schemes [8, 10–15], to our knowledge, no large-scale study has included LD-SCLC patients from an East Asian population. Therefore, we conducted a nationwide study to analyze the efficacy of definite CCRT in a large population of Korean patients with LD-SCLC using data from the Health Insurance Review and Assessment Service (HIRA) database. Korean health insurance covers the entire population of Korea, and HIRA provides information on healthcare services provided to the Korean population. Thus, by using the HIRA database, we could assess the entire Korean population.

## Methods

### Ethical statement

This study was approved by the Institutional Review Board of the Uijeongbu St. Mary Hospital, College of Medicine, Catholic University of Korea [UC18ZESI0145], and it conforms to the provisions of the Helsinki Declaration, as revised in 2013 (available at: https://www.wma.net/policies-post/wma-declaration-of-helsinki-ethical-principles-for-medical-research-involving-human-subjects/). The need for informed consent was waived because of the retrospective nature of the study.

### Study design and population

**Fig. 1:**
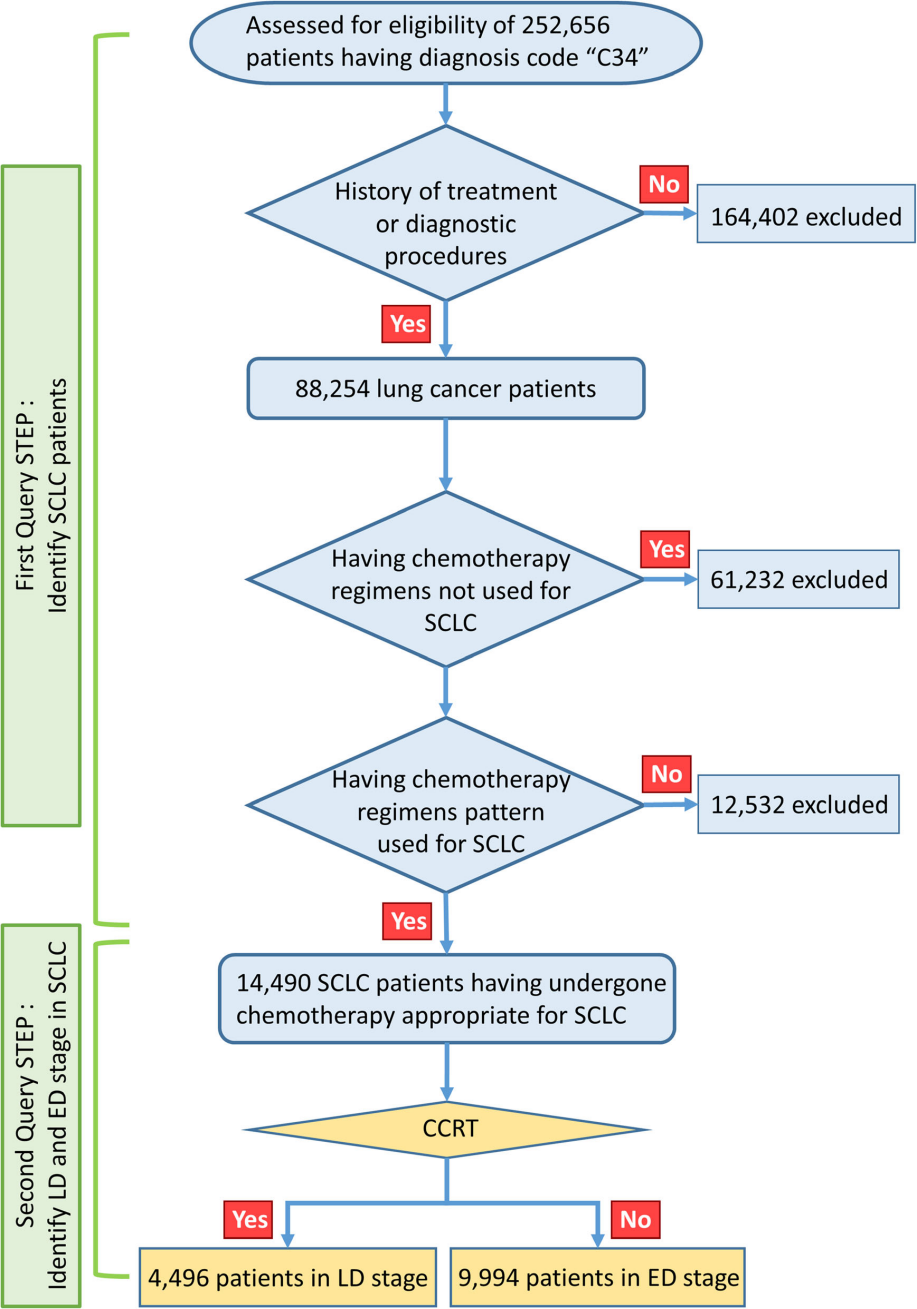
Data mining process for identification of patients with LD-SCLC. SCLC, small-cell lung cancer; CCRT, concurrent chemoradiotherapy; LD, limited-disease; ED, extensive-disease

This large-scale, retrospective, nationwide cohort study used medical insurance claim data registered in the HIRA database from January 1, 2008, to December 31, 2018, which encompassed the study period. A total of 252,656 patients with the International Classification of Diseases (ICD) code “C34” for lung cancer were identified. Among these patients, 14,490 who received chemotherapeutic regimens for SCLC under coverage by the National Health Insurance Service (NHIS) were selected, and 238,166 patients were excluded because they had received chemotherapy agents used only for NSCLC, such as paclitaxel, pemetrexed, gemcitabine, docetaxel, erlotinib, afatinib, ceritinib, crizotinib, and gefitinib. LD-SCLC was defined by a total of 20 or more lung irradiations within a total of 3 months of radiation therapy, with a total interruption period of < 30 days (Supplementary Table 1). In total, 4496 patients were identified to have LD-SCLC (Fig. 1). Of these, 4446 who received EP- or IP-based CCRT were selected for our analysis.

To verify the reliability of the operational criteria for SCLC staging, we used single-institution data from 357 SCLC patients with known disease status. Using these operational criteria, patients with LD-SCLC were predicted with a sensitivity of 64.6%, specificity of 100%, and accuracy of 88.5% (Supplementary Table 2). This operational definition facilitates identification of most cases of LD-SCLC and completely rules out ED. Thus, these definitions can be used to specifically select patients with LD-SCLC. The median survival time of LD-SCLC patients was 21.8 months (95% confidence interval [CI]: 20.86–22.96), defined as per our operational criteria, while that of ED-SCLC patients was 9.6 months (95% CI: 9.43–9.83; Supplementary Fig. 1). The corresponding 5-year survival rates were 24.73 ± 0.75% and 8.13 ± 0.30%, respectively. These findings were comparable with those for patients with LD-SCLC and ED-SCLC in recent studies [2, 16, 17]. Thus, our operational criteria were considered to be acceptable.

### Definition of survival outcomes

The primary objective was to evaluate the survival outcomes of LD-SCLC patients after CCRT. Time to first subsequent therapy (TFST) was defined as the time from the date of first-line chemotherapy to the time of subsequent chemotherapy or death from any cause, whichever was earlier. Overall survival (OS) was calculated as the time from the treatment start date to the date of death or the last follow-up visit. The date of diagnosis was defined as the date of initiation of the first chemotherapy, surgery, or radiotherapy after the first-ever application of the C34 diagnostic code. For the patients who received second-line chemotherapy, OS was calculated from the start date of the second-line chemotherapy regimen to the date of death or the last follow-up visit.

### Statistical analysis

For analysis of baseline characteristics, continuous variables were processed as means (± standard errors) or medians (range), while categorical variables were expressed as frequencies (%). A *t*-test was used for comparison of continuous variables, and Pearson’s chi-square test or the two-sample proportion z-test was used for comparison of categorical variables. Survival curves were generated using the Kaplan–Meier method and compared using the log-rank test. Unfortunately, the proportional hazards assumption was violated in this study. Poisson regression analysis, a type of generalized linear models, was performed to estimate relative risk (RR). The SAS Enterprise Guide version 6.1 (SAS Inc., Cary, NC, USA), Python 3.74 (Python Software Foundation), Visual Basic for Applications 7.0 (Microsoft Inc., Redmond, WA, USA), and Excel 2010 (Microsoft Inc.) were used for all data mining and statistical analyses.

## Results

### Baseline characteristics

A total of 4446 patients were included in our study. The baseline characteristics of the patients are summarized in Table 1. The mean age was 64 years, and 85.4% of patients were male. In total, 2187 (49.2%) patients were young (< 65 years) while 2259 (50.8%) were elderly (≥65 years). A total of 4187 (94.2%) patients received EP-CCRT and 259 (5.8%) patients received IP-CCRT. Second-line treatment was received by 1740 patients due to failure of definite CCRT or progression during treatment, 925 (53.2%) patients received combination chemotherapy, and 815 (46.8%) received single-agent chemotherapy.

**Table 1:** Demographic characteristics of 4446 patients with limited-disease small-cell lung cancer who received definite concurrent chemoradiotherapy

Variables	LD stage (*n* = 4446)
Age, years	64 ± 8.28
< 65	2187 (49.2)
≥65	2259 (50.8)
Sex (male/female)	3797 (85.4)/649 (14.6)
Comorbidities	
HBP	2361 (39.3)
DM	1334 (22.2)
Hypercholesterolemia	2314 (38.5)
First-line therapy	
Combination CRT	
Etoposide/platinum	4187 (94.2)
Irinotecan/platinum	259 (5.8)
Second-line therapy	
Combination therapy	
Etoposide/platinum	151 (8.7)
Irinotecan/platinum	774 (44.5)
Single-agent therapy	
Etoposide	1 (0.1)
Irinotecan	232 (13.3)
Belotecan	342 (19.6)
Topotecan	240 (13.8)

Values are presented as mean ± standard deviation or number (%)

*LD,* limited disease; *HBP,* hypertension; *DM,* diabetes mellitus; *CRT,* chemoradiotherapy

### Clinical outcomes of definite CCRT and second-line treatment

**Fig. 2:**
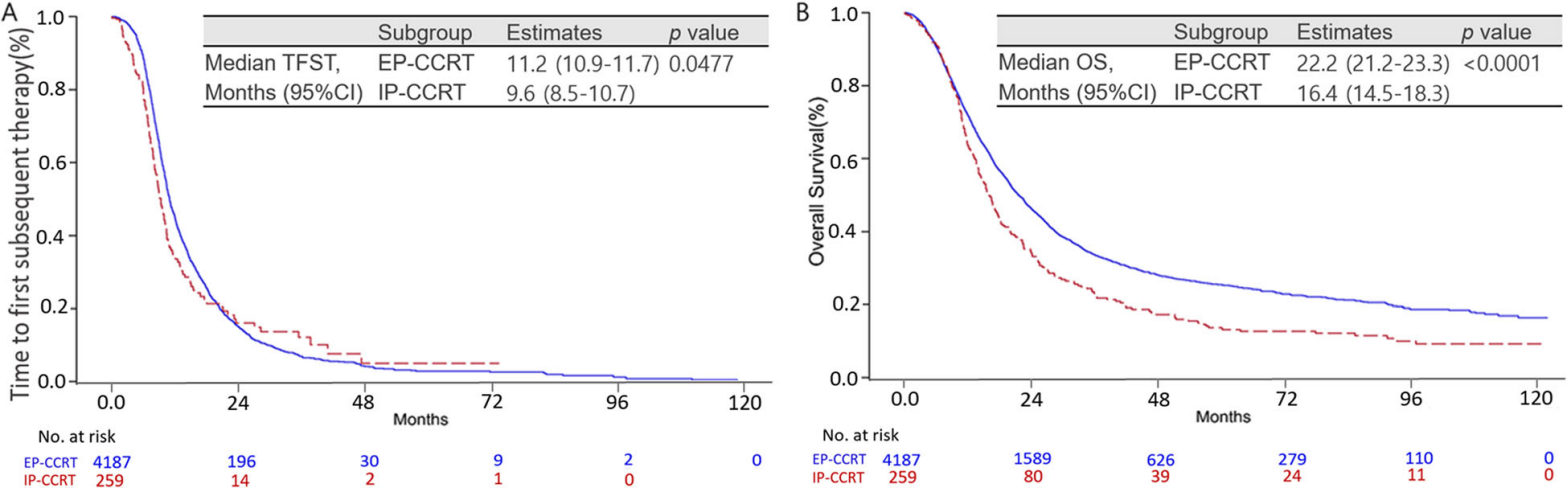
**a** Kaplan–Meier curves for time to first subsequent therapy (TFST) and **b** overall survival (OS) in patients who received an etoposide/platinum (EP)- or irinotecan/platinum (IP)-based concurrent chemoradiotherapy regimen as definite treatment for limited-disease small-cell lung cancer. TFST, time to first subsequent therapy; EP, etoposide/platinum; IP, irinotecan/platinum CCRT, concurrent chemoradiotherapy; CI, confidence interval

**Fig. 3:**
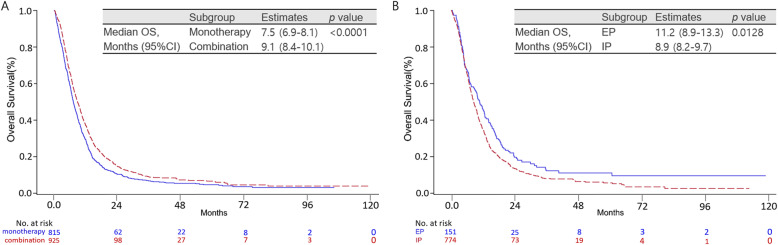
**a** Kaplan–Meier curves for overall survival (OS) in patients with limited-disease (LD) small-cell lung cancer (SCLC) who received combination chemotherapy or monotherapy (mono) as second-line treatment. **b** Kaplan–Meier curve for OS in patients with LD-SCLC who received the irinotecan/platinum (IP) or etoposide/platinum (EP) combination as second-line treatment. EP, etoposide/platinum; IP, irinotecan/platinum; CI, confidence interval

Median TFST was significantly longer with EP-CCRT (11.2 months, 95% CI: 10.90–11.67) than with IP-CCRT (9.6 months, 95% CI: 8.50–10.67; *P* = 0.0477; Fig. 2a). OS was also significantly longer with EP-CCRT (22.2 months, 95% CI: 21.23–23.33) than with IP-CCRT (16.4 months, 95% CI: 14.47–18.33; *P* <  0.0001; Fig. 2b). Among patients who received second-line chemotherapy, those who received combination chemotherapy showed a better survival benefit (9.1 months, 95% CI: 8.40–10.10) than those who received monotherapy (7.5 months, 95% CI: 6.93–8.13; *P* <  0.0001; Fig. 3a). Among combination chemotherapy regimens, the EP combination resulted in significantly better OS (11.2 months, 95% CI: 8.87–13.27) than the IP combination (8.9 months, 95% CI: 8.23–9.67; *P* = 0.0128; Fig. 3b). However, there were no significant differences in OS among single-agent regimens used for second-line treatment (*P* = 0.3712; Supplementary Fig. 2).

### Clinical outcomes of second-line treatment according to TFST of < 6 months or > 6 months after CCRT

Patients who received second-line treatment were classified into platinum-resistant and platinum-sensitive relapsed groups according to TFST of < 6 months and > 6 months after CCRT, respectively. In the platinum-resistant relapsed group, second-line IP-based chemotherapy resulted in significantly better OS (9.6 months, 95% CI: 8.67–10.33) than did second-line EP-based chemotherapy (7.1 months, 95% CI: 5.10–10.40; *P* = 0.0170; Supplementary Fig. 3A). Of note, patients who received combination chemotherapy showed a better survival benefit (9.3 months, 95% CI: 8.40–10.20) than those who received monotherapy (7.9 months, 95% CI: 7.30–8.63; *P* = 0.0100; Supplementary Fig. 3B). Conversely, second-line EP-based chemotherapy (OS: 17.2 months, 95% CI: 13.03–25.63) was superior to second-line IP-based chemotherapy (OS: 6.6 months, 95% CI: 5.43–7.77; *P* <  0.0001; Supplementary Fig. 3C) in the platinum-sensitive relapsed group.

### Factors associated with survival outcomes in patients with LD-SCLC

The results of poisson regression analyses of relative risk factors for poor survival in patients with LD-SCLC are listed in Table 2. Patients who have older age, male sex, the absence of hypertension, the absence of hypercholesterolemia, and the use of IP-CCRT as definite treatment were significantly associated with higher RR for survival than those who did not. Risk factors that were significantly associated with poor survival outcomes included older age (adjusted RR: 1.18, 95% CI: 1.12–1.22; *P* <  0.0001), the absence of hypercholesterolemia (adjusted RR: 1.32, 95% CI: 1.27–1.37; *P* <  0.0001), and the use of IP-CCRT as definite treatment (adjusted RR: 1.49, 95% CI: 1.18–1.92; *P* <  0.0001).

**Table 2:** Risk factors for overall survival in 4496 patients with limited-disease small-cell lung cancer

	Unadjusted RR		Adjusted RR	
	RR (95% CI)	*P*-value	RR (95% CI)	*P*-value
Age, years (≥65 vs. < 65)	1.15 (1.11–1.20)	< 0.0001	1.18 (1.12–1.22)	< 0.0001
Sex (male vs. female)	1.06 (1–1.12)	0.07	1.04 (0.98–1.10)	0.22
HBP (HBP vs. normal)	0.96 (0.93–1.00)	0.05	1.02 (0.98–1.06)	0.45
DM (DM vs. normal)	0.97 (0.93–1.02)	0.22	1.04 (1.00–1.09)	0.06
Hypercholesterolemia (No vs. yes)	1.28 (1.23–1.33)	< 0.0001	1.32 (1.27–1.37)	< 0.0001
Definite CCRT (IP-CCRT vs. EP-CCRT)	1.61 (1.30–2.04)	< 0.0001	1.49 (1.18–1.92)	< 0.0001

*RR*, relative risk; *CI*, confidence interval; *HBP*, hypertension; *DM*, diabetes mellitus; *CCRT*, concurrent chemoradiotherapy; *IP*, irinotecan/platinum; *EP*, etoposide/platinum

## Discussion

In the present population-based study, we investigated the efficacy of definite CCRT using real-world data for patients with LD-SCLC. The results revealed that the survival outcomes of patients who received EP-CCRT were better than those of patients who received IP-CCRT. Among second-line treatment in cases of CCRT failure or progression during treatment, the EP regimen resulted in better OS than the IP regimen; however, the IP regimen was more effective in patients with TFST of < 6 months after CCRT (i.e. the platinum-resistant relapsed patients). This study provides evidence that the EP combination should be the gold standard for CCRT in Korean patients with LD-SCLC. To the best of our knowledge, this analysis included the largest study population to date.

In a recent randomized trial including a Korean population, IP-based chemotherapy had significantly favorable effects on survival outcomes when compared to EP-based chemotherapy for previously untreated ED-SCLC [17]. Similar findings were reported in a meta-analysis of 6 trials involving 1476 patients [18]. In addition to having cytotoxic effects in SCLC, irinotecan is known to be a potent radiosensitizing agent [19], and the IP combination has been adopted as the chemotherapy regimen in CCRT for LD-SCLC. Although it has been shown that IP-based chemoradiotherapy is effective and tolerable in Asian and Western populations, there has been no direct comparison between EP-CCRT and IP-CCRT. Table 3 summarizes the results of previous studies on EP-CCRT and IP-CCRT for LD-SCLC, and shows that the efficacy of IP-CCRT was comparable to that of EP-CCRT in patients with untreated LD-SCLC. The results of phase II trials showed that median survival with IP-based CCRT was 12.4–44.5 months, which was comparable to the survival time with EP-CCRT; however, there were considerable discrepancies in results among different studies [4, 8, 10–15, 20]. In a phase II study in Japan, IP-based chemotherapy with concurrent split-course radiotherapy showed a remarkable survival benefit, with a median time to progression of 14.5 months and a median OS duration of 44.5 months [11]. However, in a phase II trial in Korean patients with LD-SCLC, the median OS was 20.0 months, with 1-year and 2-year OS rates of 85 and 35%, respectively [20]. Similarly, in a Western phase II trial, the overall radiographic response rate was 67%, the median OS was 19 months, and the 1-year and 2-year OS rates were 60 and 44%, respectively [10].

**Table 3:** Summary of previous studies on etoposide/cisplatin- or irinotecan/cisplatin-based concurrent chemoradiotherapy for limited-disease small-cell lung cancer

Author	Phase	No. of patients	Regimen	Radiation dose	RR (%)	PFS (m), *P*-value	OS (m), *P*-value	Grade 3/4 toxicity (%)
Faivre-Finn [4] (2017)	III	247	EP-CCRT BID vs. QD	45 Gy vs. 66 Gy		15.4 vs. 13.4; HR = 1.12; p = 0. 26	30 vs. 25; HR = 1.18, p = 0·14; 2 year SR (56% vs. 51%)	Neutropenia (74% vs. 65%) Esophagitis (19% vs. 19%) Radiation pneumonitis (3% vs. 2%)
Kubota [12] (2014)	III	281	EP-CCRT BID followed by IP vs. EP	45 Gy		12 vs. 13.2; HR 1.10; *p* = 0.74	33.6 vs. 38.4; HR = 1·09, p = 0·70; 5-year SR (33.7% vs. 35.8%)	Neutropenia (95% vs. 78%) Anemia (35% vs. 50%) Diarrhea (2% vs. 1%)
Fukuda [11] (2012)	II	34	IP-CCRT	50 Gy	100	14.3	44.5; 2- and 5-year SR (66.7, 46.1%)	Neutropenia 38%; Pneumonitis 6% Diarrhea 3%; Esophagitis 0%
Naidu [10] (2014)	II	36	IP-CCRT BID	45–54 Gy	67		19; 1-, 2-, 3-year SR (60, 44, 30%)	Symptomatic pneumonitis 0% Symptomatic esophagitis 13%
Saito [14] (2006)	II	51	EP-CCRT BID followed by IP	45 Gy	88	11.8	23; 2- and 3-year SR (49, 29.7%)	Neutropenia 88%; Infection 33% Electrolyte imbalance 20% Diarrhea 14%
Jeong [20] (2006)	II	20	IP-CCRT	50.4 Gy	85	12	20; 1- and 2-year SR (85, 35%)	Neutropenia 60%; Anemia (20%) Nausea/vomit(55%); Diarrhea (35%)
Hong [13] (2011)	II	19	IP-CCRT QD	54 Gy	89.5	7.6	12.4; 2-year SR (75.0%)	Radiation-induced pneumonitis 53% Neutropenia 32%
Sohn [8] (2007)	II	33	IP-CCRT BID	45–54 Gy	87.9	14.4	26.1; 2-year SR (54.9%)	Neutropenia 81.8%; Diarrhea 21.2% Radiation pneumonitis 9.1%
Han [15] (2005)	II	33	IP followed by EP-CCRT BID	45 Gy	97	12.9	25; 1- and 2-year SR (85.7, 53.9%)	Neutropenia (68% + 100%)^a^, Febrile neutropenia (20% + 60%)^b^

*RR,* response rate; *PFS,* progression-free survival; *OS,* overall survival; *EP,* etoposide/platinum; *IP,* irinotecan/platinum; *CCRT,* concurrent chemoradiotherapy; *BID,* bis in die (twice a day); *QD,* quaque die (once a day); *HR,* hazard ratio; *SR,* survival rate

^a^Grade 3 or 4 neutropenia occurred during induction chemotherapy in 68% patients and during CCRT in 100% patients

^b^Grade 3 or 4 febrile neutropenia occurred during induction chemotherapy in 20% patients and during CCRT in 60% patients

These substantial differences in efficacy among studies of IP-based CCRT may be explained by differences in the timing of radiotherapy, the optimal dose, fractionation of thoracic radiotherapy, and consolidation chemotherapy. Pneumonitis and neutropenia were the main toxicities caused by IP-CCRT. Of note, pneumonitis is an important problem associated with IP-based chemoradiotherapy [21]. A study by Ohe et al. showed that pulmonary fibrosis identified on plain chest X-rays was a strong risk factor for thoracic radiotherapy-related death [22], and that CCRT-related deaths occurred in 25 of 926 (2.7%) patients, including 7 (28%) with radiation pneumonitis [22]. In the real-world setting, the rate of poor prognostic factors, the proportion of elderly patients, and the presence of comorbidities may be higher. Furthermore, patient compliance may be worse, and routine medical practice may differ from protocol-specific patient care provided in clinical trials [23]. In the present study, patients who received EP-CCRT showed significantly better TFST and OS than those who received IP-CCRT. Thus, treatment-related toxicities, including pulmonary toxicities, may have an unfavorable impact on the patients’ clinical outcomes. Taken together, these findings suggest that the EP regimen should be strongly considered as a concurrent chemotherapeutic regimen in definite CCRT for LD-SCLC.

Concerning the strategy of using cytotoxic chemotherapy as a second-line treatment for LD-SCLC, there is no consensus on the most effective regimen. In the present study, patients who received combination chemotherapy as a second-line treatment showed significantly better OS than those who received single agents as second-line treatment, a finding that is consistent with that of a previous study [24]. Multi-agent chemotherapy has historically demonstrated response rates higher than those shown by single-agent chemotherapy in cases of relapsed SCLC. However, these better rates have often been achieved with an increase in toxicities. A recent Japanese phase III trial demonstrated the superiority of the cisplatin, irinotecan, and etoposide combination over topotecan alone as a second-line regimen for platinum-sensitive relapsed SCLC (OS: 18.2 vs. 12.5 months, HR: 0.67, 95% CI: 0.51–0.88; *P* = 0.0079) [24]. However, it has been emphasized that this combination should be administered only in select patients, such as those with platinum-sensitive relapsed SCLC, because of associated toxicities.

Clinically, the differentiation of platinum-sensitive and platinum-resistant SCLC patients is essential. Although there are some reports on the association between the platinum sensitivity status and clinical outcomes in patients with relapsed SCLC, the role of the platinum sensitivity status remains controversial [25]. In a previous study, cross-administration of chemotherapy was effective in the platinum-resistant relapsed group [25]. In the present analysis, we were able to confirm that IP-based chemotherapy resulted in better OS than did EP-based chemotherapy in platinum-resistant relapsed patients, defined by TFST of < 6 months after CCRT.

Poor prognostic factors for patients with LD-SCLC included older age, male sex, the absence of hypercholesterolemia, and the use of IP-CCRT as definite treatment. Many studies have sought to determine the changes in survival in patients with SCLC and to identify disparities between race, sex and age [26, 27]. There are many conflicting reports about the prognostic role of lipidemia in cancer patients. Some studies have shown that malignant aerobic glycolysis, or the Warburg effect, leads to efficient biomass synthesis, including lipid synthesis, which is required for malignant cell proliferation [28]. In contrast, adipose tissue modulates the storage of extrinsic potential carcinogens such as benzo(a) pyrene, which induces DNA adduct formation and prevents the accumulation of carcinogen-DNA adducts in target organs, resulting in reduced cancer risk [29]. Hypocholesterolemia is often observed in patients with advanced-stage cancer, probably because of increased demand for cholesterol by neoplastic cells, which results in increased low-density lipoprotein cholesterol removal. This dynamic is involved in the prognosis of SCLC, regulating the metabolism of lipids as dynamic organelles [30].

This study has several limitations that must be considered when interpreting the results. First, several potential biases might exist. The HIRA data were retrospectively analyzed, and there was no information regarding the radiation dose, chemotherapy dose intensity, frequency of adverse treatment-related reactions, and causes of death; these did not allow us to evaluate the prognostic roles in the treatment timing of radiotherapy [31] or prophylactic cranial irradiation. Second, identification of LD-SCLC or ED-SCLC patients as per the operational definition may be associated with bias. The sensitivity of this criterion was only 64.6%, which is a significant limitation of this study. However, to overcome such bias, we used a strict multistep approach, and the Kaplan–Meier curves for LD-SCLC and ED-SCLC showed results similar to those in recent studies. Moreover, the clinical implications were validated through single-institute pooled analysis.

The results of this study suggest that the effects of EP-CCRT on OS and TFST are significantly more favorable than those of IP-CCRT in Korean patients with LD-SCLC. In cases where CCRT fails, combination chemotherapy provides a better survival benefit than does single-agent chemotherapy. Moreover, in CCRT failure cases with TFST of < 6 months (platinum-resistant relapsed patients), IP-based chemotherapy has significantly better effects on OS and TFST than does EP-based chemotherapy.

## Supplementary Information

**Additional file 1: S1 Table.** Operational definitions used in this study. ^a)^ Chemotherapy suitable for small-cell lung cancer. **S2 Table.** Reliability of the operational criteria used for small-cell lung cancer staging. LD-SCLC, limited-disease small-cell lung cancer; ED-SCLC, extensive-disease small-cell lung cancer; OD, operational definition; PPV, positive predictive value; NPV, negative predictive value

**Additional file 2: S1 Fig.** Kaplan–Meier curve for overall survival in patients with extensive-disease (ED) and limited-disease (LD) small-cell lung cancer who received systemic treatment.

**Additional file 3: S2 Fig.** Kaplan–Meier curve for overall survival (OS) in patients with limited-disease small-cell lung cancer who received single-agent chemotherapy as second-line treatment. CI, confidence interval

**Additional file 4: S3 Fig.** Kaplan–Meier curve for overall survival (OS) in (A and B) platinum-resistant relapsed and (C) platinum-sensitive relapsed patients with limited-disease small-cell lung cancer who received the irinotecan/platinum (IP), etoposide/platinum (EP) combination or monotherapy as second-line treatment. EP, etoposide/platinum; IP, irinotecan/platinum; CI, confidence interval

## Data Availability

The datasets generated during and/or analysed during the current study are not publicly available due to Data Protection Laws and Regulations in Korea, but final analyzing results are available from the corresponding authors on reasonable request.

## Abbreviations

SCLC: Small-cell lung cancer
LD: Limited disease
CCRT: Concurrent chemoradiotherapy
EP: Etoposide/platinum
IP: Irinotecan/platinum
TFST: Time to first subsequent therapy
PFS: Progression-free survival
OS: Overall survival
ED: Extensive-disease
HIRA: Health Insurance Review and Assessment Service
ICD: International Classification of Diseases
NHIS: National Health Insurance Service
CI: Confidence interval
HR: Hazard ratio
