# Supplementary material for: Efficacy of concurrent chemoradiotherapy for patients with limited-disease small-cell lung cancer: a retrospective, nationwide, population-based cohort study
Source: BMC Cancer. 2021 Mar 31;21:340. doi: 10.1186/s12885-021-08082-2 (PMC8011172; doi:10.1186/s12885-021-08082-2)
Supplement: Supplementary file 1 — Additional file 1: S1 Table. Operational definitions used in this study. a) Chemotherapy suitable for small-cell lung cancer. S2 Table. Reliability of the operational criteria used for small-cell lung cancer staging. LD-SCLC, limited-disease small-cell lung cancer; ED-SCLC, extensive-disease small-cell lung cancer; OD, operational definition; PPV, positive predictive value; NPV, negative predictive value [file 12885_2021_8082_MOESM1_ESM.docx]

**S1 Table.** Operational definitions used in this study

| Variable | Operational definition | |
| --- | --- | --- |
| Chemotherapy^a)^ | | Etoposide or irinotecan with or without platinum as first-line chemotherapy |
| Definite of concurrent Chemoradiotherapy | | Total 20 or more lung irradiations within a total of 3 months of Radiation therapy, with a total interruption period of <30 days |

^a)^ Chemotherapy suitable for small-cell lung cancer

**S2 Table**. Reliability of the operational criteria used for small-cell lung cancer staging

|  | LD-SCLC (Real) | ED-SCLC (Real) | *P*-value | Sensitivity | Specificity | PPV | NPV | Relative risk |
| --- | --- | --- | --- | --- | --- | --- | --- | --- |
| LD-SCLC (OD) | 75 | 0 | <0.0001 | 0.6466 | 1 | 1 | 0.8546 | 6.878 |
| ED-SCLC (OD) | 41 | 241 |  |  |  |  |  |  |

LD-SCLC, limited-disease small-cell lung cancer; ED-SCLC, extensive-disease small-cell lung cancer; OD, operational definition; PPV, positive predictive value; NPV, negative predictive value
