# Supplementary figures and images for: Efficacy of concurrent chemoradiotherapy for patients with limited-disease small-cell lung cancer: a retrospective, nationwide, population-based cohort study
Source: BMC Cancer. 2021 Mar 31;21:340. doi: 10.1186/s12885-021-08082-2 (PMC8011172; doi:10.1186/s12885-021-08082-2)

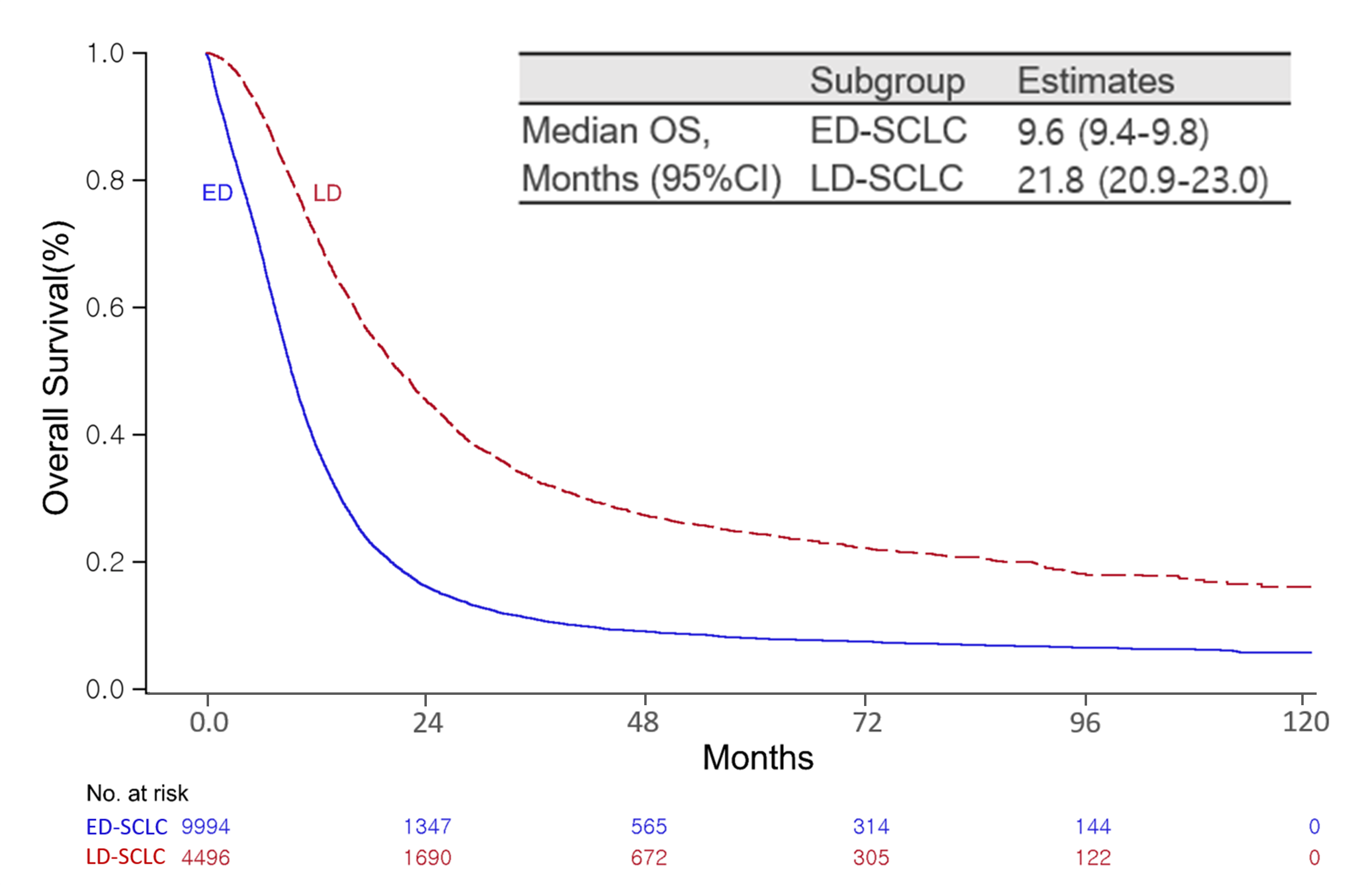

Supplement: Supplementary file 2 — Additional file 2: S1 Fig. Kaplan–Meier curve for overall survival in patients with extensive-disease (ED) and limited-disease (LD) small-cell lung cancer who received systemic treatment. [file 12885_2021_8082_MOESM2_ESM.tif]

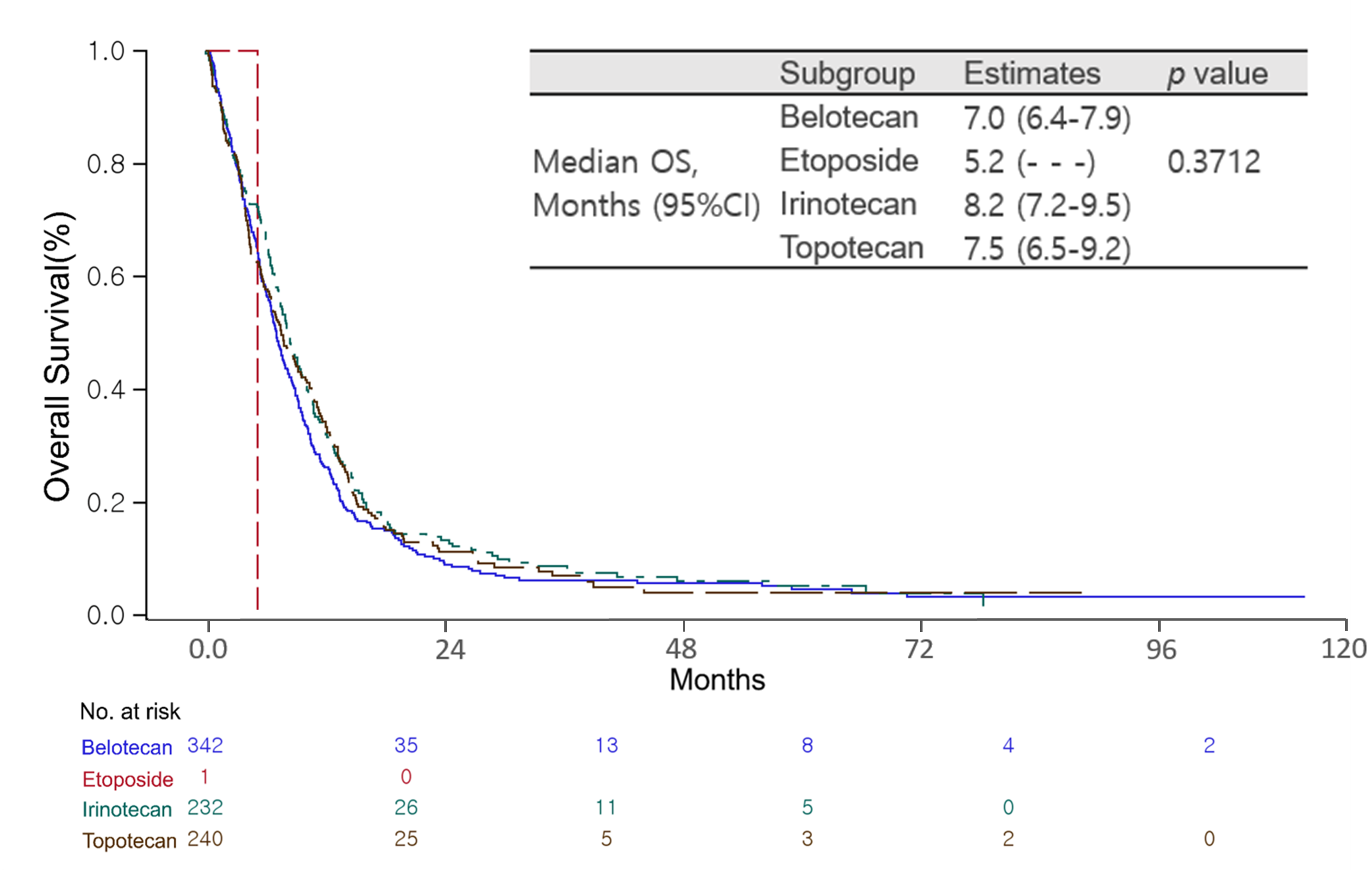

Supplement: Supplementary file 3 — Additional file 3: S2 Fig. Kaplan–Meier curve for overall survival (OS) in patients with limited-disease small-cell lung cancer who received single-agent chemotherapy as second-line treatment. CI, confidence interval [file 12885_2021_8082_MOESM3_ESM.tif]

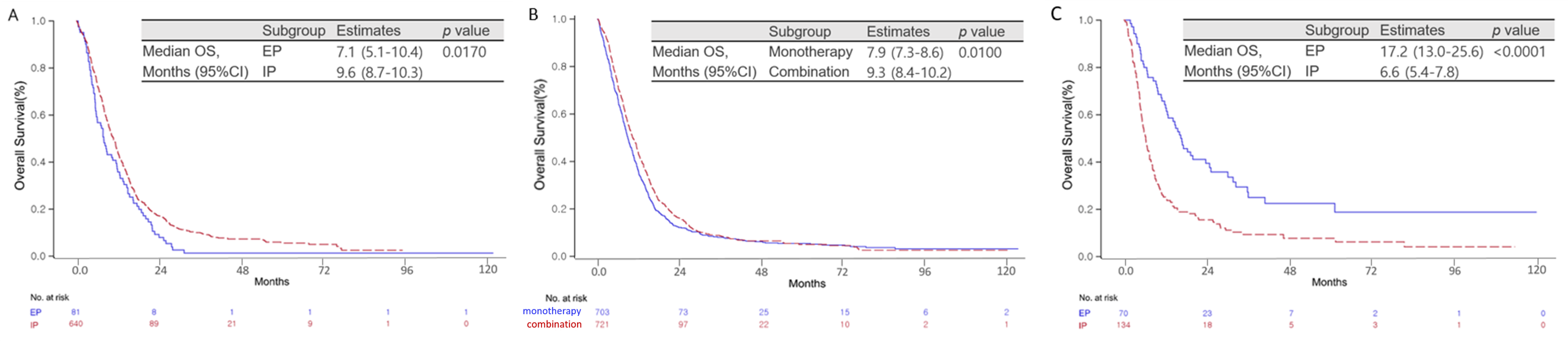

Supplement: Supplementary file 4 — Additional file 4: S3 Fig. Kaplan–Meier curve for overall survival (OS) in (A and B) platinum-resistant relapsed and (C) platinum-sensitive relapsed patients with limited-disease small-cell lung cancer who received the irinotecan/platinum (IP), etoposide/platinum (EP) combination or monotherapy as second-line treatment. EP, etoposide/platinum; IP, irinotecan/platinum; CI, confidence interval [file 12885_2021_8082_MOESM4_ESM.tif]
